# The impact of COVID-19 on health of the older persons in Bangladesh

**DOI:** 10.1007/s42379-021-00095-5

**Published:** 2021-11-02

**Authors:** Mohammad Mainul Islam, Shafayat Sultan, Mohammad Bellal Hossain

**Affiliations:** Department of Population Sciences, University of Dhaka, Dhaka, 1000 Bangladesh

**Keywords:** COVID-19, Health, Older persons, Geriatrics, Bangladesh

## Abstract

The COVID-19 is impacting the health of the population, including older persons. Available evidence shows that older people are highly vulnerable and more likely to have adverse health outcomes. In Bangladesh, the older population is rapidly increasing, living with various disadvantaged socio-economic conditions, including inadequate access and healthcare services. These disparities are likely to increase during the COVID-19, resulting in high morbidity and mortality among them. Thus, we have examined the health vulnerabilities of older persons due to the COVID-19 pandemic using content analysis. We have analyzed 102 content collected from various online and printed articles published in newspapers, journals, and other relevant sources. The study has found increased health risks, deteriorated mental health, and poor health system functioning during the pandemic and its impact on older persons in Bangladesh. Strengthening health systems through an integrated model with capacity development of existing health care providers to deal with elderly health problems, including mental health and psychosocial wellbeing; promoting preventive measures, facilitating access to healthcare is required. Bangladesh can learn the Chinese experience to adopt innovative, specialized, and advanced systems to efficiently fight against the COVID-19.

## Background

In Bangladesh, the older population is rapidly increasing, living with various disadvantaged socio-economic conditions, including inadequate access and healthcare services. Here the proportion of the older population (aged 60 years and above) has increased to 8.3% in 2021 from 6.7% in 2010 and projected to reach 12% by 2030 and 22% by 2050 (BBS, 2021; United Nations, 2019). Addressing the health vulnerabilities of these older persons is a critical concern. Multimorbidity among the older population is alarmingly high, affecting their physical capacity and daily living activities (Hossain et al., 2019). Most older persons are dying and suffering from non-communicable diseases such as heart diseases, brain stroke, high blood pressure, diabetes, kidney diseases, gastric, low vision, blood pressure, arthritis, urinary incontinence, cataracts, etc. (BBS, 2021; DPS, 2019). Older women have a higher prevalence of diseases compared to men (DPS, 2019). The major problems are identified as lack of health literacy, lack of health services and social support, and lack of healthcare-seeking behaviors (Rahman et al., 2021a, 2021b, 2021c). Here older people are heavily dependent on their family members for seeking healthcare-related services. In this regard, different personal barriers such as financial hardship, lack of support from family members, as well as institutional barriers such as lack of older people friendly mechanisms in the health care service centers, high cost of health care related services, mismanagement regarding the supply of services are creating barriers against proper health care utilization of older people in the context of Bangladesh (DPS, 2019).

Thus the burden of disease among the elderly population has been increased overtimes. The health service delivery system -an intricate web of public health departments, NGOs, and private institutions has not been ready at all to address the elderly health problems in the country. The health system's readiness to deal with the elderly health issues in terms of access to health care, management process, and wellbeing of the older population is not well enough. There are no age-friendly hospital services in different health facilities; only one tertiary level hospital has a geriatric unit. However, only 3% of health care providers have specialized training to deal with geriatric problem facilities (Rahman, 2020). Geriatric health problems are highly prevalent in the country, whereas institutional and social capacities to address the same remain limited (Hossain et al., 2020; Mazumder et al., 2020). The essential component of the health structure of a country is the availability of health care services and making health care services accessible to the general people. A significant portion of older persons is not getting treatment for the morbidities. More or less 30% of the older adults do not receive any treatment for morbidities irrespective of gender or residence (BBS, 2015). Moreover, health care facilities for the elderly are scarce and mainly by private entrepreneurship (BBS, 2015).

In Bangladesh the presence of higher malnourishment and diverse disability among older people is also prominent (Alam et al., 2021; Rahman et al., 2021a, 2021b, 2021c). Regarding the mental health of older persons, the country has been reported as having a very high prevalence of geriatric depressive symptoms (Rahman et al., 2020a, 2020b, 2020c), which is often connected to the malnourishment and other health-related problems of older persons (Rahman et al., 2021a, 2021b, 2021c). Though the concern is serious, geriatric mental health is given one of the lowest priorities (Alam et al., 2021). Inadequate management of these health problems increases the risks of disability and poor quality of life among the older population (Uddin et al., 2017).

Given the above-mentioned health vulnerabilities of the older persons, the COVID-19 pandemic is creating enormous challenges in Bangladesh. Amidst the coronavirus period, The Government of Bangladesh adopted different non therapeutic measures which included declaring nationwide holiday with the closure of all government and private offices, shutting down all academic institutions, cancelling international and reducing national flight schedules, restricting the movements of mass vehicles and posing transportation restrictions, red flagging the houses of returnees who have returned from countries having cases of coronavirus, deploying law enforcement authorities to ensure the implementation of travel and mobility restrictions etc.

Like many countries, Bangladesh also went for shut down in March 2020 to reduce COVID-19 infection in the communities. The quick adoption of lockdown measures may change the health care seeking behavior and support networks overnight. In addition, the country has adopted social distancing as one of the public health measures to reduce COVID-19 transmissions in the community. Such measures could have adverse effects on the mental health of the population. Older adults are the most at-risk group for COVID-19 because their mortality risk increases with age, particularly those living with chronic conditions. In Bangladesh, COVID-19 fatalities were higher among older adults. In addition, older persons experienced many struggles over testing for COVID-19 because of the lack of institutional and structural supports like lack of testing booths for older people, transportation difficulties, and mobility problems by within and outside the health systems (Islam et al., 2021a, 2021b).

The lockdown measure urged people aged 60 years and above to avoid social contact and stay home. Such measures can have direct and indirect effects on older persons' health and psychosocial wellbeing. Older adults who were previously mobile but are housebound now are at risk of developing psychological infirmity through disruption of balance and home confinement. During this COVID-19, all these become a public health threat to older adults. Under the lockdown situation there can be presence of functional limitations, absence of home support services and aged residential care and health professionals to support older adults living in the community. Thus, this study, along with the pre-existing and emerging new vulnerabilities, aims to track the impact of COVID-19 on older adults' health issues and health services utilization in Bangladesh.

## Data and Methods

This study has utilized content analysis (CA) as a design as it allows the analysis of content collected from written, verbal, visual materials from printed, electronic, and digital sources. We have performed searches in public domains like google, google scholar, and PubMed, both non-academic and academic databases commonly used to explore newspaper articles and literature reviews.

In this content analysis (CA), we analyzed 102 pieces of content from various online and printed newspapers, opinion/advice/advocacy articles, articles published in different national and international journals, situation assessment reports, and other relevant sources published in both English and Bengali between March 1, 2020 and May 15, 2021. We have chosen this point of time because the first coronavirus case was detected in Bangladesh on March 8, 2020. The types and numbers of content explored as a part of CA are given below (Table 1).

**Table 1:** Types of Contents explored as a part of Content Analysis (March 1, 2020–May 15, 2021)

Types of content	No. of news/Findings explored
Newspaper articles	74
Opinion articles	11
Data/study published in the journals	11
Situation assessment reports	3
Others	3
Total	**102**

While exploring the content, we have made sure that the contents specifically included the focus on issues of the population of interest (older persons in this case), the validity of the evidences (by cross checking with other contents especially in the cases of daily newspapers), and absence of inconsistency. Moreover, while selecting the sources, specifically in the cases of newspaper articles, we selected newspapers with most coverage, credibility and reputation.

We analyzed the data using thematic approach. Salient themes were constructed based on the recurrence of the issues explored from content analysis. After that, the constructed themes were organized and interpreted in the findings section keeping the main aim of the paper ahead. Relevant verbatim was provided with each theme in the findings section. In addition, a brief comparative discussion of Bangladesh’s older population with the impact of COVID-19 on the health of the older Chinese population has been provided.

## Findings and discussions

The content analyses explored different dimensions of the health of older persons in Bangladesh, which COVID-19 and its associated issues impacted. Different themes regarding COVID-19 imposed health risks for older persons, consequences regarding mental health issues, heavy burden on health systems and health care structures during COVID-19 and its effect on older population's health care access, utilization, and health outcome etc. were emerged through the content analysis. The findings are discussed below.

### The increased health risks of COVID-19 for older persons in Bangladesh

The age factor, which exposes older persons worldwide to the high stakes of death due to COVID-19, was similar for Bangladesh. A large share of total death due to COVID-19 occurred in Bangladesh's age category of 60 and above. The altered lifestyle pattern due to various restrictions also increased the other health risks for older persons. In the state-imposed lockdown phases, the limits in usual activities were creating barriers towards maintaining the health of the older persons in Bangladesh.

### Disruption of health enhancing routine physical activities

The fear of getting infected with the COVID-19 decreased the frequency of health-enhancing routine physical activities of older persons in Bangladesh. The disruption was also caused by the mobility restrictions (Chowdhury, 2020). An urban woman aged 73 years of Dhaka city, stated her reasons behind decreasing physical health-enhancing activities during COVID-19 to BBC News Bangla -

“Earlier, I used to go for a walk both in the morning and evening, but now, I fear the risks of Coronavirus. I am scared to even touch anything without washing my hands. I keep rubbing my hands with hexasol. I will surely die if it comes into my body. Even the developed countries are going through the hardest struggles. So it's better to be safe and not move anywhere.” (Chowdhury, 2020)

### Increased health risks due to presence of multimorbodity

The presence of high level of multimorbidity among older population is increasing the risks of COVID-19 in Bangladesh. These concerns were reflected in different news and media reports. Health service providers perceived the increased health risks regarding older persons in Bangladesh during this COVID-19 period considering the present burden of multimorbidity. A physician of Bangabandhu Sheikh Mujib Medical College University, stating his opinion regarding the challenging situation of older persons in Bangladesh during COVID-19 to the online newspaper ‘Jugantor’.

“In our country, different diseases like diabetes, hypertension, and asthma are very common among people 50 years and older. The presence of this multimorbid situation decreases their lung's working capacity. And this multimorbidity pattern is worsening their ability to cope up with the diseases. So, they are dying in a large number in Bangladesh." ("Older persons are at greater risk of Coronavirus, the things must be considered,” 2020)

### Compromised health care seeking pattern of older population

According to the different published news and media reports, the healthcare-seeking pattern of older persons also influenced the rise of the risks. The late visits of older persons to different health care facilities, despite showing COVID-19 related symptoms in this pandemic period, increased their chances of death. This is because the older persons living with comorbidities were comparatively much vulnerable than any other population group; hence they required quick management of health care services after being tested positive with COVID-19 or after starting to show COVID-19 related symptoms. But in Bangladesh, the tendency of holders regarding late visits towards health care facility centers was reported mainly. It was considered one of the crucial reasons behind their increased risk of death in this pandemic situation. A high official of Directorate General of Health Services, explained the reason behind the increased death risk of older persons in Bangladesh during the COVID-19 period to the online newspaper ‘Dhaka Today’-

“If we analyze the death rate, we see that the largest portion of people who died from Coronavirus was from old age category. People living with comorbidities like cancer, diabetes, high blood pressure, asthma are more likely to die. Many of the cases among those who died were so late to visit hospitals that physicians could not do anything. After the failure of all organs, they came to seek care. But at that time, there was nothing to do.” (Islam, 2020, 2020a, 2020b, 2020c)

Perceived insusceptibility and lack of seriousness regarding the disease severity made older people less willing to pay a timely visit to health care centers even after being tested positive with COVID-19. At the same time, lack of trust towards the health care services and health care management of different service centers also increased their hesitancy to receive COVID-19 related services timely. A high official of Directorate General of Health Services, explained the reason of unwillingness of older persons to receive health care services in Bangladesh during COVID-19 period to the online newspaper ‘Dhaka Today’ -

“Lots of beds are still empty. We want older persons to come here as soon as they are detected with Coronavirus. But they are not coming. One reason can be their lack of awareness. But at the same time, it is also true that they lack trust in health care services. This is also creating unwillingness among them.” (Islam, 2020, 2020a, 2020b, 2020c)

### Higher health risks of Rohingya older population

The risks of getting infected with COVID-19 and other associated health risks were even higher among the older persons living in the Rohingya refugee camps in Bangladesh. The lack of information about the ways of spread, preventive practices, potential consequences, and the overcrowded housing patterns left them in a very risky context. The least inclusion of older refugee persons in the humanitarian response plan was reported to be worsening the situation in this regard. A high official from Amnesty International's Crisis Response opined about the vulnerability of older refugee persons in the Rohingya communities during the COVID-19 pandemic period-

“Repeating this same mistake amid the COVID-19 pandemic puts older Rohingya women and men in imminent danger—with some of them not even receiving the most basic information about what is happening and how they can best stay safe.” (Amnesty International UK, 2020)

The earlier faults in recognizing older persons' right to health, food, water, and sanitation in the refugee camps were repeated even in the COVID-19 context when the older people are the most at-risk population. On top of that, fear and stigmatized attitude regarding the spread of coronavirus and preventive measures were very much prominent among the older persons in refugee camps due to the lack of information. The traditional information campaign did not work well for older persons, thus leaving them at a wider risk (Amnesty International UK, 2020). An older woman of Rohingya camp described her inability to capture the information provided about COVID-19 in the camp -

“I don't know anything about that virus, just people are saying something about a virus on the mike, but I don't hear well, that's why I don't know anything… I'm always thinking, what they keep saying.” (Amnesty International UK, 2020)

Such challenges widened the risks of older persons in Bangladesh during this pandemic period, which needed rapid and sustained initiatives. In the absence of such initiatives, the death rate and morbidity burden due to COVID-19 would be heavier in the upcoming days.

### Mental Health of Older Persons during COVID-19

In the current COVID-19 pandemic period, the older persons were challenged with the risks of getting infected and death due to COVID-19, and the present context took a toll on their mental health too, which should also be considered. Sudden changes in the daily lifestyle pattern, changes in the family, social, and community support process due to COVID-19, and facing newer preventive practices like social distancing, quarantine and isolation were the causes behind their psychological trauma during this pandemic period in Bangladesh (Azizul, 2020). In addition, the burden of psychological stress was weighted by the previous mental health issues, financial and social constraints amidst this pandemic period, fear of losing near and dear ones during COVID-19, etc.

In a context when a high prevalence of geriatric depressive symptoms in Bangladesh was already very much prominent (Rahman et al., 2020a, 2020b, 2020c), social isolation, risks of poverty during the pandemic period were worsening the scenario for older people in Bangladesh (Rahman et al., 2020a, 2020b, 2020c). Mental health issues always remained neglected in the health policy planning of Bangladesh, and not surprisingly, the geriatric mental health services also were facing different constraints amidst this pandemic period (Rahman et al., 2020a, 2020b, 2020c). Thus the susceptibility of older persons to mental health complications even increased in this pandemic period, which necessitated focused and targeted intervention for them.

### Poor functioning of the health system during the pandemic period and its impact on older persons

It was evident that the first and second wave of Coronavirus has put tremendous pressure on the health system of Bangladesh. This pressure was felt much by the older persons of the country. The pre-existing health conditions of older persons made them more likely to face serious consequences after being infected by Coronavirus. Proper health system functioning, including adequate access to intensive care facilities across the country was needed to treat the older persons in such severe circumstances. However, different media reports and news highlighted the inadequacy of such care facilities, which exposes the older persons at risks than any other population group (“Protect elderly people from coronavirus,” 2020).

### Lack of emergency and older people friendly health care facilities

The demand and supply side mismatch within the health system regarding the health care services of older persons amidst this pandemic period has been reflected in the published newspapers where it was shown that both lacks of crucial health care facilities and skilled workforce were largely responsible for the increased risks of older persons during COVID-19 waves.

"Elderly people with pre-existing health conditions such as diabetics and hypertension are very vulnerable to coronavirus infection. If they get infected, a large portion of them will need intensive care and ventilation. There is a huge gap between demand and supply in terms of intensive care facilities. ICU management in the country is very poor. ICUs in the private hospitals are not up to the mark forlack of necessary equipment and skilled workforce." ("Protect elderly people from coronavirus", 2020)

Easy access towards COVID-19 related health care services for older persons was also unavailable, especially in the earlier phase of the Coronavirus in Bangladesh, according to different news published in electronic and print media. The unavailability of provision of prioritized COVID-19 testing facilities for older persons in health care centers was reported in other media to highlight their vulnerability during this pandemic period. The complications with receiving online serial for testing were of much trouble for older persons. The long waiting line for completing the testing procedures created barriers to seeking testing-related services for older persons. An older person, described his struggle to understand the procedures for COVID-19 testing in front of a reputed tertiary health care center in Dhaka to online newspaper jagonews24.com -

"I don't understand these procedures of online tokens. I don't know now where to go and where to seek help for this! The system is too much hard for me." (Uzzal, 2020).

### Absence of home-based care amidst coronavirus

Though the absence of home-based care for older persons within the health system of Bangladesh was always a concern, it was much felt amidst this pandemic period. Nationwide travel restrictions during the lockdown phases and limited availability of transports during COVID-19 increased the challenges of older persons to access the COVID-19 specialized and other general hospitals. The absence of home-based services or specialized facilities in the nearest point of contact increased the vulnerability of older (Hossain et al., 2020).

#### Rejections by tertiary hospitals

The earlier indecisive policy approach of transformation of general hospitals into dedicated COVID-19 also caused disruptions in health care seeking process of older persons. At the earlier stage of the pandemic, different tertiary hospitals who were not yet transformed to dedicated COVID-19 hospitals, were reported to be rejecting older adults showing COVID-19 symptoms -

“……..However, there have been multiple reports of patients, despite showing symptoms of Coronavirus, being refused by different hospitals in Dhaka, including Kurmitola General Hospital. In one instance, an elderly person aged around 60 with Covid-19 symptoms died at the Shaheed Suhrawardy Medical College and Hospital early Thursday after being reportedly rejected by Dhaka Medical College Hospital (DMCH) and Kurmitola General Hospital” (Hasan, 2020)

The COVID-19 imposed a strain on the overall health system could be the reason for delaying regular outpatient visits of older persons with psychological conditions and cardiovascular diseases, which extended the risks of the severity of the general non-COVID-19 disorders as well as COVID-19 related health complications (Rahman et al., 2020a, 2020b, 2020c).

## Conclusion and implications

Bangladesh will experience very rapid growth of the older population among the South Asian countries in the next 20–30 years. With a rapid pace of growth, the older population will have different influential impacts on the country's socio-economic structure where employment generations are critical, and the dependency of the more aging population is much prominent. Therefore, ensuring the health and wellbeing of older persons will be an essential concern for the coming years. Currently, Bangladesh has been experiencing an epidemiological transition where non-communicable diseases are the major killer of death. In addition, COVID-19 has made enormous challenges for the health sector of Bangladesh. The health system is struggling to provide care to critical patients, most of whom are older persons. Here the case fatality rate indicates that older persons are dying at a higher rate than other age groups.

. The health system of Bangladesh is not ready yet to cope with the increasing demand of the older population. There is a widespread lack of trained health care providers to deal with the health and wellbeing of the older population. During this COVID-19 pandemic, the importance of developing a framework for age-friendly hospital initiatives for health services in a low-resource setting has been revealed. An integrated model with capacity development of existing health care providers to deal with elderly health problems can be useful in Bangladesh, where older persons' mental health and psychosocial wellbeing of older persons can be ensured. In this regard, strengthening health systems, promoting preventive measures, facilitating access to healthcare, and providing social care to providers and institutions to protect older persons' health and wellbeing of older persons are required. To limit the spread of infection and death rate, Bangladesh can learn the Chinese experience. We know, China, the first country affected by the COVID-19, revealed that the case fatality rate was 8% amongst 70–79 years, which was almost double among the individuals aged 80 years or more (Wu & McGoogan, 2020). Chinese evidence also suggests strategies are required to reduce the adverse psychological and occupational impacts on healthcare workers (HCWs) and the population in a lockdown reported as poor psychological wellbeing (Cheng et al., 2021; Meng et al., 2020; Zhang et al., 2021). At the beginning of the outbreak of COVID-19 healthcare system of China experienced intrinsic problems-public hospitals being overwhelmed, a dire shortage of personal protective equipment, and exhaustion of health human resources and resource costs, leading to the widespread and exponential rise of infected cases at the early stage of the epidemic (Sun et al*.*, 2021). However, China later managed to improve the situation by adopting innovative, specialized, and advanced systems, including Internet based hospitals- an innovative organizational form and service mode under the tide of internet plus in the Chinese medical industry and high technologies such as 5G, big data analysis, cloud computing, and artificial intelligence (Sun, S. et al*.* 2021). Thus, the efficient use of these new innovative technologies can help Bangladesh win its fight against the virus.
